# A reappraisal of the default mode and frontoparietal networks in the common marmoset brain

**DOI:** 10.3389/fnimg.2023.1345643

**Published:** 2024-01-09

**Authors:** Takuto Okuno, Noritaka Ichinohe, Alexander Woodward

**Affiliations:** ^1^Connectome Analysis Unit, RIKEN Center for Brain Science, Wako, Saitama, Japan; ^2^Laboratory for Ultrastructure Research, National Institute of Neuroscience, National Center of Neurology and Psychiatry, Kodaira, Japan

**Keywords:** default mode network, frontoparietal network, common marmoset, independent component analysis (ICA), fMRI, general linear model (GLM)

## Abstract

In recent years the common marmoset homolog of the human default mode network (DMN) has been a hot topic of discussion in the marmoset research field. Previously, the posterior cingulate cortex regions (PGM, A19M) and posterior parietal cortex regions (LIP, MIP) were defined as the DMN, but some studies claim that these form the frontoparietal network (FPN). We restarted from a neuroanatomical point of view and identified two DMN candidates: Comp-A (which has been called both the DMN and FPN) and Comp-B. We performed GLM analysis on auditory task-fMRI and found Comp-B to be more appropriate as the DMN, and Comp-A as the FPN. Additionally, through fingerprint analysis, a DMN and FPN in the tasking human was closer to the resting common marmoset. The human DMN appears to have an advanced function that may be underdeveloped in the common marmoset brain.

## 1 Introduction

The default mode network (DMN), a network of brain regions in humans, is activated when a person is at rest, during introspective moments like remembering the past, envisioning the future, or when considering the thoughts and perspectives of other people (Buckner and Carroll, 2007; Buckner et al., 2008). This prominent network has also been observed in other animal species such as the chimpanzee (Barks et al., 2013), macaque (Vincent et al., 2007; Mantini et al., 2011), common marmoset (Belcher et al., 2013; Liu et al., 2019), rat (Lu et al., 2012), and mouse (Stafford et al., 2014). The DMN can be extracted through several neuroimaging techniques, such as independent component analysis (ICA) of resting-state (rs-) fMRI (functional magnetic resonance imaging) (Beckmann and Smith, 2004), seed-based connectivity analysis (SCA) of rs-fMRI (Cole et al., 2010), or by task-induced deactivation of general linear model (GLM) analysis of task-fMRI (Buckner et al., 2008; Binder, 2012). Usually, the ICA approach is favored for the extraction of large brain network components in humans and non-human primates. Furthermore, task-induced deactivation is another important technique to help identify the DMN regions where it was originally observed in positron emission tomography (PET) blood flow studies (Binder, 2012). In early studies task-induced decreases in blood flow were largely ignored (Binder, 2012). However, Shulman et al. (1997) showed that task-induced decreases in blood flow were a common phenomenon in PET activation studies. Later, this phenomenon was termed the “default mode” of brain function by Raichle et al. (2001). The areas of the DMN are widely agreed upon for the human brain [see Table 1, and for example Buckner et al. (2008)]. However, for the common marmoset (Callithrix jacchus), a non-human primate, the homolog of the human DMN has been a hot topic of discussion in recent years in the marmoset research field. The common marmoset has recently had interest as an experimental animal as it is closer to the human than rodents. Therefore, the homolog of the human DMN in the marmoset has importance for the further understanding of several pathological studies related to the DMN, e.g., Alzheimer's (Xu et al., 2020).

**Table 1:** Default mode network regions under investigation and their areas.

	**Marmoset Comp-A**	**Human DMN**	**Marmoset Comp-B**
mPFC	N/A	Parts of A9, A10, A32, A11	N/A
dlPFC	Parts of A8b, A6M, A6DR	Parts of A6, A8, A9, A46	Parts of A8b, A6M, A6DC
PPC	LIP, MIP, VIP, OPt; PG As A39	A39; parts of A40, A7	PE, PF; PFG As A39
PCC	A19M, A23V, PGM	A23, A31	A23a, A23b, A31
Temporal	N/A	A21, A22	N/A

The DMN of the common marmoset was first described by Belcher et al. (2013). Group-ICA was applied to rs-fMRI sessions, and was defined as consisting of the retro-splenial and posterior cingulate cortex (PCC) region (A23, A31, A29, and A30 areas), the dorsolateral prefrontal cortex (dlPFC) region (A6DR, A6DC, and A8C areas), the posterior parietal cortex (PPC) region surrounding PE, PFG, PG, and the left intraparietal sulcus (LIP) and middle intraparietal sulcus (MIP). Ghahremani et al. (2017) identified the same network component by group-ICA, but they instead defined it as the frontoparietal network (FPN). This was because it had previously been reported and identified as a frontoparietal network controlling saccades in resting-state network (RSN) studies of anesthetized macaques (Hutchison et al., 2011). Liu et al. (2019) refuted this and argued that this component is the DMN, because it was found that task-induced deactivation in visual-task fMRI occurs around the PCC (PGM and A19M areas) and PPC (LIP and MIP areas) regions. This definition was continued with in Tian et al. (2022). In later research, Hori et al. (2020) applied fingerprint analysis (Passingham et al., 2002) using several sub-cortical regions and found that this component was the closest to the DMN component obtained from human rs-fMRI, and therefore concluded it to be the DMN of the common marmoset. Ngo et al. (2023) applied joint gradient analysis (Xu et al., 2020), and gradient 2 showed similarity between the resting human DMN and marmoset dlPFC-PCC-PPC network. Although these studies appear to have reached some consensus, some studies continue to use the FPN definition (Schaeffer et al., 2019; Garin et al., 2022). Furthermore, there remains a large mismatch between functional and structural investigations. Some functional studies (Belcher et al., 2013; Liu et al., 2019; Hori et al., 2020; Ngo et al., 2023) support the DMN definition of PCC (PGM and A19M areas) and PPC (LIP and MIP areas) for the common marmoset, but neuroanatomical (cytoarchitectonic) results (Hutchison et al., 2011; Ghahremani et al., 2017) do not support it as the homolog of the human DMN.

**Figure 1:**
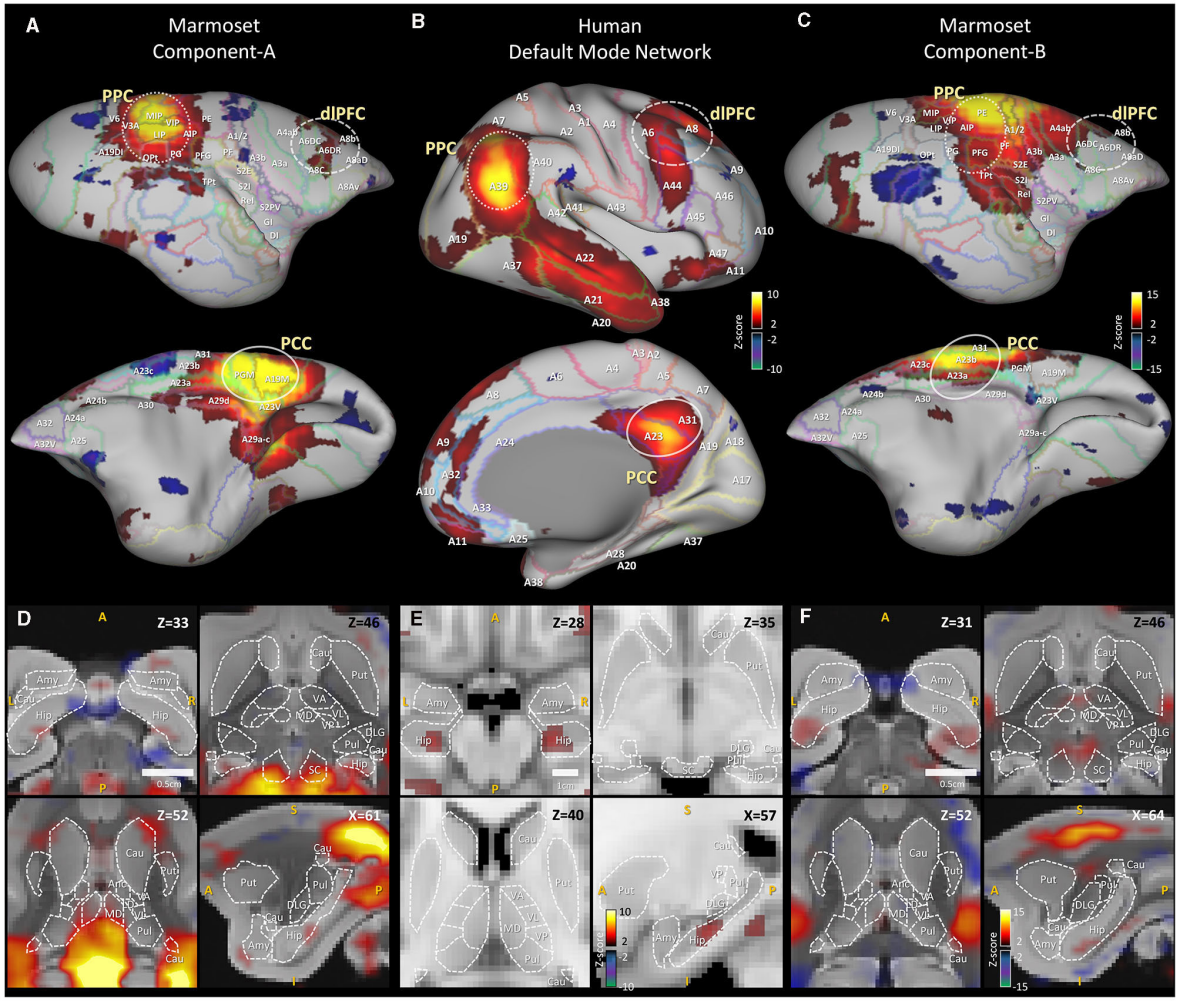
Human default mode network component and awake marmoset ICA components. **(A)** Right cortical surface of the marmoset, (top) lateral side, (bottom) medial side. Awake resting-state marmoset ICA component-A, selected from 30 components, mapped onto the brain surface. Z-score range is 2 to 15 for positive, −2 to −15 for negative. **(B)** Right cortical surface of the human brain. Human resting-state default mode network is mapped onto the surface. Z-score range is 2 to 10 for positive, −2 to −10 for negative. **(C)** Right cortical surface of the marmoset brain. Awake resting-state marmoset ICA component-B, mapped onto the brain surface. **(D)** Horizontal views (top left, right and bottom left) and a sagittal view (bottom right) of awake marmoset ICA component-A. Scale bar shows 0.5 cm. Z-score range is from 2 to 15. **(E)** Horizontal views (top left, right and bottom left) and a sagittal view (bottom right) of human resting-state default mode network component. Z-score range is from 2 to 10. Scale bar shows 1 cm. **(F)** Horizontal views (top left, right and bottom left) and a sagittal view (bottom right) of awake marmoset ICA component-B. Cau, Caudate; Put, putamen; Hip, hippocampus; Amy, amygdala; SC, superior colliculus; Anc, thalamus anterior nuclear complex; LD, laterodorsal; MD, mediodorsal; VA, ventral anterior; VL, ventral lateral; VP, ventral posterior; Pul, pulvinar; DLG, lateral geniculate.

In this study, we carefully restarted from a neuroanatomical point of view and identified two ICA components (Comp-A and Comp-B) as candidates for the DMN. Component-A (Comp-A) is the (earlier described) network that in the literature has been called either the DMN, or FPN in the common marmoset. Comp-A peaks at Paxinos's LIP and MIP areas (of the PPC), and PGM and A19M areas (of the PCC) (Table 1, Figure 1A). Another one, Component-B (Comp-B), has previously been called the somatomotor network (SMN) in the common marmoset (Belcher et al., 2013; Hori et al., 2020). It peaks at the PE area (of the PPC), and A23b and A31 areas (of the PCC) (Table 1, Figure 1C). We next reviewed Liu et al.'s visual-task fMRI experiment and noticed that their marmosets were trained to reduce their saccades. In the human case, the saccade task showed a significant BOLD signal decrease in the intraparietal sulcus which includes the LIP, MIP areas (DeSouza et al., 2003). Thus, we performed GLM analysis with a more appropriate auditory-task fMRI (Binder et al., 1999; Crone et al., 2011) dataset to check for deactivated regions in the marmoset cortex. We confirmed the anatomical connectivity (from retrograde tracing) between the medial prefrontal cortex (mPFC) region (A10 area) and PCC region (A23 and A31 area) and evaluated their functional connectivity through multiseed-based connectivity analysis. Finally, we performed fingerprint analysis [following Hori et al. (2020)] by using several sub-cortical regions. We made comparisons of marmoset fMRI not only with human resting-state fMRI network components, but also with human task-fMRI [working memory (wm)-task and motor-task] network components. Through these analysis results we propose that Comp-A is the FPN and Comp-B is the DMN of the common marmoset. We also found that the resting marmoset's Comp-A and Comp-B were closer to the wm-task human components than the resting human components. This suggests that the marmoset may not be resting like humans do during fMRI experiments, or, based on the combination of this result and multiseed-based connectivity analysis between mPFC and PCC regions, the resting-state DMN may be underdeveloped in the common marmoset brain.

## 2 Methods and materials

### 2.1 Preprocessing of marmoset resting-state fMRI data

Awake resting-state fMRI data of the common marmoset (*Callithrix jacchus*) were acquired as part of the Brain/MINDS project (Okano et al., 2015; Muta et al., 2023). A Bruker BioSpec 9.4T MRI machine (Biospin GmbH, Ettlingen, Germany) was used. The experimental settings of the gradient recalled echo planar imaging (EPI) sequence were as follows: flip angle = 65, repetition time (TR) = 2,000 ms, echo time (TE) = 16 ms, pixel size = 0.7 × 0.7 mm, slice thickness = 0.7 mm, matrix size = 60 × 42 × 52, and frame length = 150.

For our experiments, T1WI, T2WI, and rs-fMRI NIfTI files of awake marmosets (3 to 6 years, 3 males and 1 female, 12 sessions per subject) (*N* = 48) were used. Preprocessing and image registration were performed using Statistical Parametric Mapping (SPM12) (Penny et al., 2011). Realignment was applied for NIfTI images to compensate for head movement by a least squares approach and a 6 parameter (rigid body) spatial transform. Slice timing correction was performed to correct for signal acquisition timing discrepancies in each section, and images were co-registered to the Marmoset MRI Standard Brain (Iriki, 2017). We removed the first 10 frames of the rs-fMRI data, and the remaining data were smoothed using a full width at half maximum (FWHM) of 1.4 mm (2 voxels) for group ICA [for compatibility with Liu et al. (2019)]. A FWHM of 2.4 mm (3.4 voxels) was used for multiseed-based connectivity and fingerprint analysis. Global mean (the average signal across all voxels) and aCompCor (Behzadi et al., 2007) were applied for nuisance factor removal and a high-pass filter (1/128Hz) was applied for subsequent analyses.

### 2.2 Independent component analysis of marmoset resting-state fMRI data

After preprocessing, independent component analysis (ICA) was applied to the marmoset rs-fMRI data to acquire 30 components. The number of components was chosen for compatibility with (Liu et al., 2019). MELODIC (Beckmann and Smith, 2004) was used to obtain group ICA from 48 sessions (140 frames). Here, multi-session temporal concatenation was performed and a spatial map was obtained. Finally, the two components that were used in our study, Comp-A and Comp-B, were manually selected from the 30 components.

For surface mappings of marmoset data, we first converted NIfTI images from the Marmoset MRI Standard Brain space (Iriki, 2017) to the Marmoset Brain Mapping V3 space (Liu et al., 2021). Then, “wb_command -volume-to-surface-mapping,” included in the Connectome Workbench visualization software (Marcus et al., 2011), was used to map NIfTI image data onto the marmoset cortical surface. Finally, the cortical surface (in gray), the functional data mapped to the surface, and the Paxinos label map (Paxinos et al., 2012) were overlaid to produce our figures.

### 2.3 Multiseed-based connectivity analysis of marmoset resting-state fMRI data

Multiseed-based connectivity analysis was done by calculating the correlation coefficients between seed voxels and all other voxels. MATLAB scripts for this analysis were developed in-house and worked together with the VARDNN toolbox (Okuno and Woodward, 2021). To investigate the functional connectivity between the frontal pole and PCC regions, the seed voxels of the marmoset mPFC and PCC regions were manually edited in ITK-SNAP (Yushkevich et al., 2006). After calculating the correlation coefficients in each voxel from individual sessions, a mixed-effects model was applied to acquire final group results. To elaborate, an averaging of single-subject correlation is a fixed-effects model and its fixed value will be specific to the sampling group data used. Going further, a mixed-effects model estimates a whole group value by a statistical test. A one-sample *t*-test in each voxel was performed for 2nd-level (group) analysis (Holmes and Friston, 1998). Bonferroni correction was then applied to correct for the familywise error (FWE) rate and *t*-value threshold (*t* > 6.48 in **Figure 3**) were applied to acquire significantly correlated voxels.

### 2.4 Fingerprint analysis of marmoset resting-state fMRI data

Fingerprint analysis (Passingham et al., 2002) was used to analyze the correspondence between marmoset and human ICA components. To apply fingerprint analysis, we used 14 sub-cortical regions as fingerprints (Supplementary Figure 1) from the Brain/MINDS 3D Marmoset Reference Brain Atlas 2019 (Woodward, 2019). The correlation between component time-series (resting marmoset FPN/DMN) and voxel time-series in sub-cortical regions was calculated for all marmoset sessions. A mixed-effects model was applied for group analysis and the *t*-value of each voxel was calculated by one-sample *t*-test. Mean *t*-values were used to quantify the fingerprints of the 14 sub-cortical ROIs. Finally, the Manhattan distance between resting marmoset FPN/DMN and resting/task human FPN/DMN components was calculated using all 14 fingerprints.

### 2.5 Preprocessing of marmoset auditory task-fMRI data

Gilbert et al. (2023) performed an auditory task-fMRI experiment with the common marmoset. We used their auditory task-fMRI data to investigate task-induced deactivation. Three functional time courses were acquired from two awake marmosets (named M3 and M4). Details of the data are orientation: axial, resolution: 500-μm isotropic, FOV: 48 × 48 mm, number of slices: 42, number of volumes: 205, TE: 15 ms, BW: 400 kHz, flip angle: 40°, acceleration rate: 2 (left-right).

T2WI and task-fMRI NIfTI files (*N* = 6) were used for registration. Preprocessing and registration were performed using Statistical Parametric Mapping (SPM12) (Penny et al., 2011). SPM12 registered NIfTI images to the Marmoset MRI Standard Brain (Iriki, 2017), and task-fMRI data was smoothed using a FWHM of 1.7 mm (3.4 voxels). The preprocessed task-fMRI data was then used for GLM analysis.

### 2.6 GLM analysis of marmoset auditory task-fMRI data

GLM analysis was used to investigate the details of the task-induced deactivation. The canonical haemodynamic response function (HRF) used for GLM analysis was characterized by two gamma functions with peak time around 3.1 s (for the marmoset) (Yen et al., 2018). A simple GLM design matrix was used with one variable, four nuisance variables and an intercept. Data for the first variable were created by convolution of the canonical HRF from block car designs corresponding to sound stimuli. Data for the four nuisance variables were calculated from the average values for each time point of the white matter, CSF, all brain voxels, and the average signal over all voxels. A high-pass filter (1/128Hz) was applied to the target variable and first variable, then a Tukey taper (taper size = 8) was used for GLM pre-whitening (Woolrich et al., 2001). The mixed-effects model was used for group analysis and the *t*-value of each voxel was calculated by 2nd-level analysis of OLS regression with a Tukey taper. Then, we applied a voxel-wise primary threshold (uncorrected *p* < 0.001 and *t* > 4.14) to obtain significantly activated or deactivated voxels (Woo et al., 2014), and a cluster-extent threshold (k > 69 voxels and FWE corrected *p* < 0.049) was applied to acquire significant clusters under multiple comparisons.

### 2.7 Preprocessing of HCP resting-state fMRI data

Resting-state fMRI data from the WU-Minn HCP consortium [the S500 release (Van Essen et al., 2013)] were used for our experiments. Scanning used a customized SC72 gradient insert and a body transmitter coil with 56 cm bore size, and data was saved in NIfTI format. Experimental settings of the gradient-echo echo-planar imaging (EPI) sequence were as follows: flip angle = 52, repetition time (TR) = 720 ms, echo time (TE) = 33.1 ms, pixel size = 2 × 2 mm, slice thickness = 2 mm, matrix size = 104 × 104 × 90, multiband factor = 8, and frame length = 1,200. More information on the resting-state parameters can be found at the HCP website: (https://www.humanconnectome.org/storage/app/media/documentation/s500/HCP_S500_Release_Reference_Manual.pdf).

T1WI, T2WI, and rs-fMRI NIfTI files from the S500 release were downloaded and a total of 200 sessions (50 male subjects × 2 sessions, 50 female subjects × 2 sessions) were used in our experiments. The CONN toolbox (Whitfield-Gabrieli and Nieto-Castanon, 2012) was used for preprocessing. CONN performed the realignment and co-registration of NIfTI images to the standard Montreal Neurological Institute (MNI) brain space. The first 10 frames of the rs-fMRI data were removed and the remaining data were smoothed using a FWHM of 4 mm (2 voxels) for group ICA [for compatibility with Liu et al. (2019)], and a FWHM of 6.8 mm (3.4 voxels for compatibility with the marmoset data) for multiseed-based connectivity and fingerprint analysis. Global mean and aCompCor (Behzadi et al., 2007) were applied for nuisance factor removal and a high-pass filter (1/128Hz) was then applied for subsequent analyses.

### 2.8 Preprocessing of HCP task-fMRI data

Three types of task-fMRI data (working memory, motor, social) were obtained from the WU-Minn HCP consortium [the S500 release (Van Essen et al., 2013)]. We chose these data because the motor task is a very basic task for fMRI studies, the social task was used in previous studies for the marmoset (Liu et al., 2019; Gilbert et al., 2021), and we assumed the wm-task deactivates the DMN. The experimental settings of the EPI sequence were the same as for the resting-state fMRI data. T1WI, T2WI, and rs-fMRI NIfTI files from the S500 release were downloaded and a total of 200 sessions (100 male subjects, 100 female subjects) were used in our experiments. The CONN toolbox (Whitfield-Gabrieli and Nieto-Castanon, 2012) was used for task-fMRI data preprocessing. CONN registered NIfTI images to the standard Montreal Neurological Institute (MNI) brain space. Data were smoothed using a FWHM of 4 mm (2 voxels) for group ICA, and a FWHM of 6.8 mm (3.4 voxels for compatibility with the marmoset data) for multiseed-based connectivity and fingerprint analysis. A high-pass filter (1/128Hz) was applied for subsequent analyses.

### 2.9 Independent component analysis of HCP resting/task fMRI data

After preprocessing, group ICA was applied to acquire 15 components from the human rs-fMRI data. We systematically checked several different numbers of components - 5/10/15/20/30 - and decided that 15 components were appropriate. For example, the default mode network became separated into two components if 30 components were chosen. MELODIC (Beckmann and Smith, 2004) was used to obtain group ICA from 200 sessions. Here, multi-session temporal concatenation was performed and a spatial map was obtained. Finally, the DMN, FPN and SMN components used in our study were manually selected from the 15 resting/task fMRI data components.

For surface mappings of human data, the command “wb_command -volume-to-surface-mapping” of the Connectome Workbench visualization software (Marcus et al., 2011) was used to map NIfTI image data onto the human cortical surface. Finally, the cortical surface (in gray), the mapped functional data, and the Brodmann label mapping (included in the HCP data) were overlaid to produce our visualizations.

### 2.10 Multiseed-based connectivity analysis of HCP resting/task fMRI data

The procedure of multiseed-based connectivity analysis of HCP resting/task fMRI data was the same as for the marmoset. A *t*-value threshold (*t* > 5.96 in **Figures 3**, **5**) was applied to acquire significantly correlated voxels.

### 2.11 Fingerprint analysis of HCP resting/task fMRI data

Fingerprint analysis (Passingham et al., 2002) was used to analyze the correspondence between marmoset and human ICA components. To apply fingerprint analysis, we used 14 sub-cortical fingerprints (Supplementary Figure 1) from the ALLEN HUMAN REFERENCE ATLAS - 3D, 2020 (Ding, 2020) for the human data. The correlation between component time-series (resting or task human FPN/DMN) and voxel time-series in sub-cortical regions was calculated for all data. The procedure to acquire *t*-values of 14 sub-cortical ROIs was the same as for the marmoset. In each ROI, non-parametric Steel-test and Bonferroni correction was performed to test the difference of the mean value of the voxel *t*-value distributions for the resting marmoset vs. resting/wm-task human (FPN/DMN). Finally, the Manhattan distance (Hori et al., 2020) between resting marmoset FPN/DMN and resting/task human FPN/DMN/SMN components was calculated using the fingerprints of the 14 sub-cortical ROIs. The permutation test was applied to test the significance of the Manhattan distance. *t*-values of 14 sub-cortical ROIs were permutated in each species, and 240,000 distances were calculated. A two-sided rank test was performed and Bonferroni correction was applied to the results.

### 2.12 GLM analysis of HCP task-fMRI data

The design matrix for GLM analysis was composed of several contrast variables, four nuisance variables and an intercept. Data for the contrast variables were created by convolution of the canonical HRF with block car designs corresponding to task stimuli. Data for the four nuisance variables were calculated from the average values at each time point of the white matter, CSF, all brain voxels, and all voxels of the volume. A high-pass filter (1/128Hz) was applied to the target and contrast variables, then a Tukey taper (taper size = 8) was used for GLM pre-whitening (Woolrich et al., 2001). The mixed-effects model was applied for group analysis and the *t*-value of each voxel was calculated by a 2nd-level analysis of OLS regression with a Tukey taper. We applied a voxel-wise primary threshold (uncorrected *p* < 0.001 and *t* > 3.10) to obtain significantly activated or deactivated voxels (Woo et al., 2014), and a cluster-extent threshold (k > 55 voxels and FWE corrected *p* < 0.049) was applied to acquire significant clusters under multiple comparisons.

### 2.13 Statistical information

For multiseed-based connectivity analysis, a mixed-effects model was used for group analysis. A one-sample *t*-test for each voxel was performed as a 2nd-level (group) analysis. Statistical significance was set at *p* < 0.05. Bonferroni correction was then applied to correct for the familywise error (FWE) rate and a *t*-value threshold was applied to acquire significantly correlated voxels.

For GLM analysis, a mixed-effects model was used for group analysis and the *t*-value of each voxel was calculated by 2nd-level analysis of OLS regression with a Tukey taper. We applied a voxel-wise primary threshold (uncorrected *p* < 0.001 and *t* > 4.14) to obtain significantly activated or deactivated voxels, and a cluster-extent threshold (k > 69 voxels and FWE corrected *p* < 0.049) was applied to acquire significant clusters for the HCP task-fMRI data. For the marmoset task-fMRI data, a voxel-wise primary threshold (uncorrected *p* < 0.001 and *t* > 3.10) and a cluster-extent threshold (k > 55 voxels and FWE corrected *p* < 0.049) were applied.

## 3 Results

### 3.1 Anatomy-based comparison of DMN regions

Figure 1 shows the results of our anatomy-based comparison. The human DMN component (Figures 1B, E) is visualized in between two awake marmoset ICA components (Comp-A and Comp-B) (Figures 1A, D, C, F). To acquire the human DMN component, 200 sessions of HCP rs-fMRI data (Van Essen et al., 2013) were pre-processed by the CONN toolbox (Whitfield-Gabrieli and Nieto-Castanon, 2012), and group ICA (MELODIC Beckmann and Smith, 2004) was applied to acquire 15 components from the human rs-fMRI data. The DMN component was then manually selected. For the awake marmoset ICA components, rs-fMRI data were acquired as part of the Brain/MINDS project (Okano et al., 2015; Muta et al., 2023) and pre-processed by Statistical Parametric Mapping (SPM12) (Penny et al., 2011). Then, 30 components were acquired by group ICA in the same manner as for the human components. The PCC region of the human DMN component peaks around Brodmann's (Van Essen et al., 2013) A23 and A31 areas (Figure 1B), however, Comp-A, which was previously called the marmoset DMN or FPN, peaks at Paxinos's (Paxinos et al., 2012; Liu et al., 2021) PGM and A19M areas on the marmoset cortex (Figure 1A). In neuroanatomical terms, these are inconsistent results. The PPC is also inconsistent: Comp-A peaks at Paxinos's LIP and MIP areas, but a previous study in the macaque monkey showed that the LIP receives input from many visual areas (Lewis and Van Essen, 2000) and has direct neural connections to the frontal eye field (FEF) and the superior colliculus (SC), which are the center of the saccade oculomotor system (Schall et al., 1995; Stanton et al., 1995). Figure 1D showed strong positive Z-score in SC area (top right), but the human case did not (Figure 1E top right). The MIP of the macaque monkey also seems to closely resemble the function of the human medial intraparietal cortex (Grefkes and Fink, 2005). This is why Comp-A has been repeatedly called the FPN. The PPC of the human DMN component peaks around Brodmann's A39 area (Figure 1B), which corresponds to the vicinity of the PG and PFG areas (De Schotten et al., 2012; García et al., 2014) of the marmoset cortex. We systematically examined the different components generated by ICA and found what we call Comp-B to have neuroanatomically (cytoarchitectonic) better fitting regions with the human DMN. Comp-B has a peak around the A23b, A31 areas for PCC, and includes the PG, PFG areas rather than MIP, LIP for PPC (Figure 1C). Comp-B peaks at the PE area, which would correspond to Brodmann's A7 area of the human cortex, which is dorsal to the human A39 area, and also includes parts of the A1, A2, and A3b areas, which are related to somatosensory function. Although Comp-B did not have positive Z-score areas in the temporal lobe and mPFC regions, which are positive in the human DMN component, Comp-A also did not have peaks around them. Comp-B also shows overlapped areas in the PCC and PPC of the DMN regions based on architectonic analysis, therefore it could be a fascinating DMN candidate. In a previous study, this component was called the (dorsal medial) somatomotor network (SMN) (Belcher et al., 2013; Ghahremani et al., 2017). However, it peaks around the PE and A23b, A31 areas, but not A4ab [primary motor and somatosensory areas (Cléry et al., 2020)]; we therefore think this component does not match with the SMN.

### 3.2 Task-induced deactivation of DMN regions

**Figure 2:**
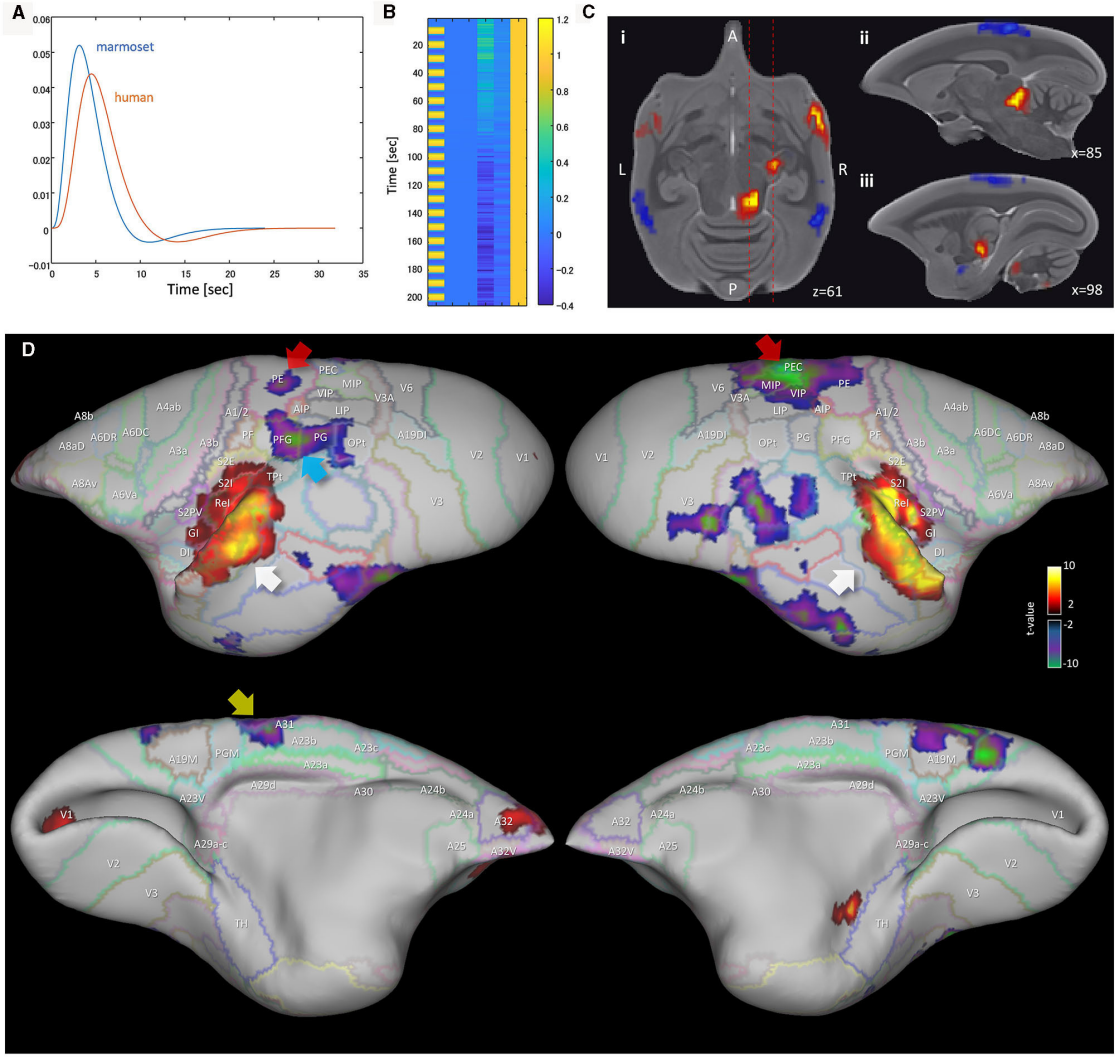
GLM analysis results of awake marmoset passive auditory task-fMRI. **(A)** Canonical hemodynamic response function (HRF) for the marmoset and human. **(B)** Example design matrix for GLM analysis (TR = 3 seconds). **(C)** GLM analysis result (auditory stimuli > rest) of sub-cortical regions. (i) Horizontal plane (z = 61) of marmoset brain shows several activated regions. (ii) Sagittal plane (*x* = 85) shows activated region of inferior colliculus. (iii) Sagittal plane (*x* = 98) shows activated region of medial geniculate nucleus. **(D)** GLM analysis result (auditory stimuli > rest) mapped to the marmoset cortical surface. White arrow shows activated region of auditory cortex. Red arrow shows deactivated region of PEC, PE. Yellow arrow shows deactivated regions of A23b, A31. Cyan arrow shows the deactivated region of PFG.

Liu et al. (2019) collected visual task-fMRI of the common marmoset and found that task-induced deactivation occurs around the PCC (PGM and A19M) and PPC (LIP and MIP) regions. However, we found that marmosets were trained to reduce their saccades and as a result the eye-tracking signal was reduced (see Figure 1 of their article). This may cause deactivation around the LIP area and may give confounding results. For the human case, the pro-saccade task showed a significant BOLD signal decrease in the intraparietal sulcus (IPS) compared to the anti-saccade task (DeSouza et al., 2003). Additionally, Gilbert et al. (2021) performed a social task-fMRI experiment with two marmosets in a whole-body human-spec 3T MRI and their marmosets were not trained to reduce saccades. Results showed activations around the PCC (PGM and A19M) and PPC (LIP and MIP) regions for both the face-to-face and movie watching paradigms. This is inconsistent with Liu et al.'s (2021) result. Therefore, we propose that an auditory-based investigation of task-induced deactivation, rather than visual-based, may be more appropriate. For the human case, a passive sentence listening task showed significant deactivation in the PCC and mPFC regions (Crone et al., 2011), and a tone discrimination task showed significant deactivation in the PCC, PPC and mPFC regions (Binder et al., 1999). Recently, Gilbert et al. performed an auditory task-fMRI experiment (a passive marmoset vocalization stimuli) with the common marmoset (Gilbert et al., 2023), but they did not visualize a surface mapping of the task-induced deactivation across the brain. We acquired their auditory task-fMRI data and performed GLM analysis to investigate the details of the task-induced deactivation (Figure 2). The human and common marmoset have different peak times in their hemodynamic response functions (HRF); 5–6 s for the human (Bonakdarpour et al., 2007) and around 3.1 s for the marmoset (Yen et al., 2018). The canonical HRF used for GLM analysis was characterized by two gamma functions. Figure 2A shows example canonical HRFs for the marmoset and the human. The design matrix for GLM analysis is simple, with one variable, four nuisance variables and an intercept (Figure 2B). Auditory stimuli minus resting contrast (auditory stimuli > rest) were used for the analysis. We could successfully reproduce the activated auditory related regions of Gilbert et al.'s (2023) result, such as the inferior colliculus, medial geniculate nucleus, and auditory cortex (Figure 2C). Figure 2D shows a surface mapping of the GLM analysis for the auditory task-fMRI, and the auditory cortex showed task-induced activation (white arrow). From this confirmation we further investigated the task-induced deactivation. The VIP, LIP, and A19M areas did not show peak deactivation during the auditory-based task (Supplementary Table 1 gives detailed voxel rates). Instead of these areas, PEC and PE (red arrow), A23b and A31 (yellow arrow), PG, PFG (cyan arrow) and part of MIP, V2 were deactivated by the task. This result is roughly consistent with task-induced deactivation in macaque monkeys (Mantini et al., 2011) in A23, A31, PEa and PGm except A24/32, A23v, A9/46d and A8b. Comp-A has a positive group ICA result in A23V, A19M, PGM, MIP, VIP, LIP, OPt, PG and part of A8b, while Comp-B is positive in A23, A31, PE, PF, PFG, A1/2, A3b and part of A8b. Although both components showed several overlapping areas between component and task-induced deactivation, A23, A31 and PE appear as consistent areas between common marmoset and macaque monkeys, therefore we prefer Comp-B as a more suitable DMN component.

### 3.3 A10 (mPFC) and A23a (PPC) have weak anatomical connectivity

**Figure 3:**
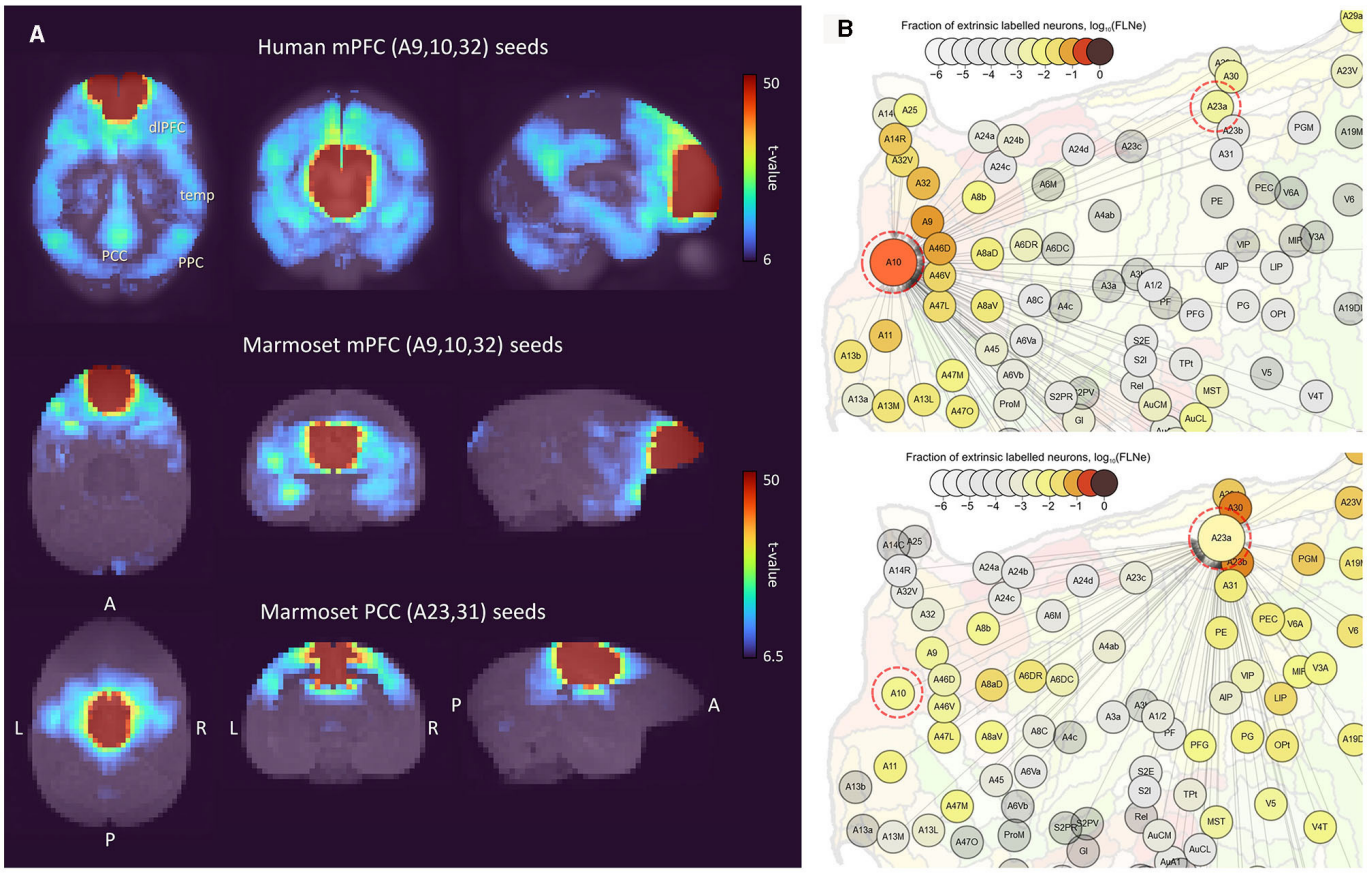
Multiseed-based connectivity analysis results of human and awake marmoset resting-state fMRI. **(A)** Three-dimensional maximum projection of t-values from multiple seeds are shown. (Top) Result for seeds in human mPFC (part of A9, A10 and A32 areas). (Middle) Result for seeds in marmoset mPFC (parts of A9, A10, and A32). (Bottom) Result for seeds in marmoset PCC (parts of A23a, A23b, and A31). **(B)** Retrograde tracing results of marmoset cortex from the Marmoset Brain Connectivity Atlas (Majka et al., 2020). (Top) Injection point in area A10. (Bottom) Injection point in area A23a.

The functional connectivity of the mPFC region of the common marmoset has been investigated in works such as Liu et al. (2019) and Garin et al. (2022). These showed that the marmoset does not have functional and anatomical connections between the PCC and PPC regions. Since we propose a new DMN candidate, Comp-B, we also investigated the functional and anatomical connectivity between DMN regions through multiseed-based connectivity analysis. Figure 3A shows analysis results for human and awake marmoset resting-state fMRI. The human mPFC (parts of A9, A10, and A32) seeds showed significant functional connectivity with other DMN regions (Figure 3A top), whereas the marmoset mPFC (parts of A9, A10, and A32) did not show significant functional connectivity at all. This result is consistent with Liu et al.'s (2019). We also checked the functional connectivity of the marmoset PCC (parts of A23a, A23b, and A31) and it did not show significant functional connectivity to the mPFC. However, based on the marmoset cortex retrograde tracing results from the Marmoset Brain Connectivity Atlas (Majka et al., 2020), anatomical connections were observed between A10 and A23a (Figure 3B), but not observed between A10 and PGM/A19M. This result also supports Comp-B as a DMN candidate.

### 3.4 Fingerprint analysis of resting and task state FPN and DMN

**Figure 4:**
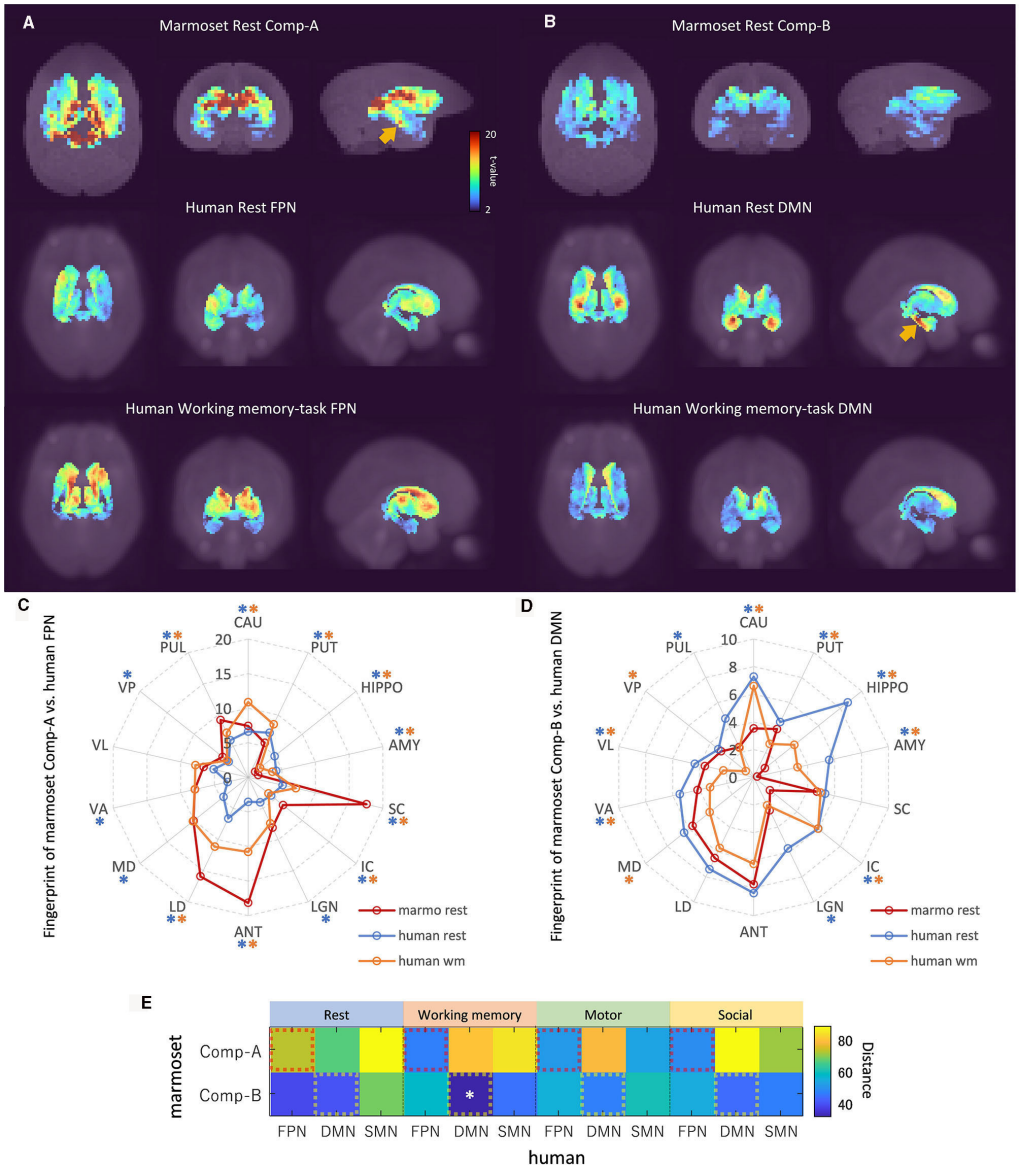
Fingerprint analysis result between awake resting marmoset and resting/tasking human ICA components**. (A)** Example fingerprint result of three-dimensional maximum projection of *t*-values. Correlation between Comp-A time-series and voxel time-series in sub-cortical regions was calculated. The closest two ICA components are shown at the top row (awake resting marmoset) and bottom row (wm-task human). The middle row shows resting human for reference. *t*-value color bar is the same for all. **(B)** Example fingerprint result of three-dimensional maximum projection of *t*-values (Comp-B). The bottom row shows wm-task human. **(C)** Radar chart of fingerprint result of 14 sub-cortex regions (resting marmoset Comp-A, resting and wm-task human FPN). Blue and orange asterisks show the significantly different *t*-values between resting marmoset vs. resting human FPN, and resting marmoset vs. wm-task human FPN (*p* < 0.05) in each ROI by the non-parametric Steel-test and Bonferroni correction. **(D)** Radar chart of DMN fingerprint result of 14 sub-cortex regions (resting marmoset Comp-B, resting and wm-task human DMN). Blue and orange asterisks show the significantly different *t*-values between resting marmoset vs. resting human DMN, and resting marmoset vs. wm-task human DMN (*p* < 0.05) in each ROI by non-parametric Steel-test and Bonferroni correction. **(E)** Fingerprint distance results between awake resting marmoset components and resting/task human components. White asterisks show the significantly close distance (*p* < 0.05) by permutation test and Bonferroni correction.

Hori et al. (2020) applied fingerprint analysis (Passingham et al., 2002) to analyze the correspondence between marmoset and human ICA components. To apply fingerprint analysis, they used 14 sub-cortical fingerprints (right hemisphere regions): Caudate (CAU); putamen (PUT); hippocampus (HIPPO); amygdala (AMY); superior colliculus (SC); inferior colliculus (IC); and a set of thalamic ROIs (regions of interest), namely, the lateral geniculate nucleus (LGN), anterior (ANT), laterodorsal (LD), mediodorsal (MD), ventral anterior (VA), ventral lateral (VL), ventral posterior (VP), and pulvinar (PUL) (Supplementary Figure 1). Topological features of sub-cortical regions are well preserved between the marmoset and human (Supplementary Figure 1), and regional functions are also assumed to be homologous among primate species. We used these same fingerprints where the sub-cortical ROIs were taken from the Brain/MINDS 3D Marmoset Reference Brain Atlas 2019 (Woodward, 2019) for the marmoset, and the ALLEN HUMAN REFERENCE ATLAS – 3D, 2020 (Ding, 2020) for the human. The correlation between component time-series (resting marmoset FPN/DMN and resting/task human FPN/DMN) and voxel time-series in sub-cortical regions was calculated for all marmoset and human sessions. A mixed-effects model was applied for group analysis and the *t*-value of each voxel was calculated by one-sample *t*-test (Holmes and Friston, 1998). Figure 4 shows the fingerprint analysis result between awake resting marmoset and resting/task human ICA components. The 3D maximum projection of *t*-values in sub-cortical voxels are shown in Figures 4A, B, with the top row showing the component-to-voxel correlation results of resting marmoset FPN/DMN components. The middle row shows the resting human FPN/DMN components, where we can see a component-to-voxel correlation difference between resting marmoset and resting human. When we used the mean *t*-values to quantify the fingerprints of the 14 sub-cortical ROIs (Figures 4C, D), the resting human vs. resting marmoset Comp-A showed significant differences in 13 ROIs, more than the 9 ROIs for the wm-task human vs. resting marmoset Comp-A (Figure 4C). This result suggests that the marmoset resting state may be closer to the human wm-task state, and the marmoset may not be “resting” like a human does during fMRI experiments.

The Manhattan distance (Hori et al., 2020) between resting marmoset FPN/DMN and resting/task human FPN/DMN/SMN components was calculated using the fingerprints of the 14 sub-cortical ROIs (Figure 4E, and the extra marmoset ICA component version is available in Supplementary Figure 3). Hori et al. (2020) showed that marmoset Comp-A was closer to the human DMN component than to the FPN or SMN components in the resting-state and our results have been consistent with theirs (Figure 4E, resting human). It is known that the hippocampus is involved in the human DMN (Figure 1E; Buckner et al., 2008), and Comp-A showed a slightly stronger correlation in the hippocampus (Figure 4A, yellow arrow). This was also shown in the resting human DMN component (Figure 4B, yellow arrow). However, if we consider the task human components, only the resting marmoset Comp-B and wm-task human DMN was found to be significantly close by permutation test and Bonferroni correction (Figure 4E). Comp-A was closer to the human FPN components than to the human DMN or SMN components in all three task states (working memory, motor, and social tasks). Furthermore, Comp-B was consistently closer to the human DMN components than to the human FPN or SMN components in the three task states. Our fingerprint analysis also indicated that the marmoset's Comp-B [named dorsal medial SMN in a previous study (Belcher et al., 2013; Ghahremani et al., 2017)], is not close to the resting/task human SMN components.

### 3.5 Analysis results of human working memory-task fMRI data

**Figure 5:**
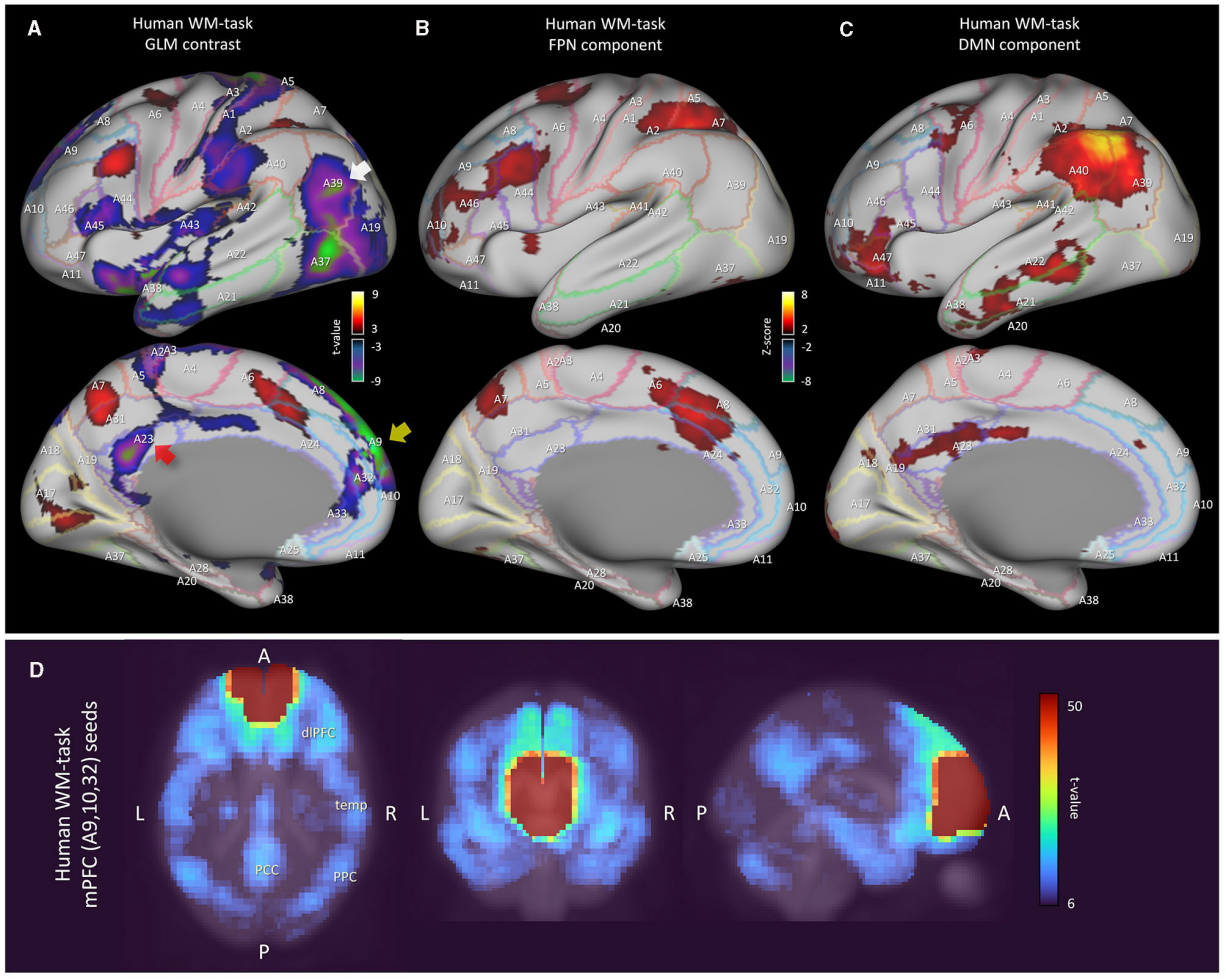
Analysis results of human working memory-task fMRI data. **(A)** Left cortical surface of the human brain. GLM result of 2-back vs. 0-back contrast mapped onto the surface. The *t*-value range is 5 to 9.1 for positive, −5 to −10.6 for negative. **(B)** Left cortical surface of the human brain. Wm-task human FPN component is mapped onto the surface. Z-value range is 3 to 15 for positive, −3 to −15 for negative. **(C)** Left cortical surface of the human brain. Wm-task human DMN component is mapped onto the surface. **(D)** Multiseed-based connectivity analysis result of human wm-task fMRI using mPFC seeds (part of A9, A10 and A32 areas). Three-dimensional maximum projection of *t*-values from multiple seeds are shown.

Finally, we investigated the human working memory task-fMRI data when compared with marmoset resting-state fMRI data (Figure 5). Within each run of the wm-task, 4 different stimulus types were presented in separate blocks. Also, within each run half of the blocks used a 2-back wm-task and half used a 0-back wm-task (as a working memory comparison) (Van Essen et al., 2013). The GLM result of 2-back vs. 0-back contrast was then mapped onto a 3D digital brain surface (Figure 5A). This task deactivated DMN regions, such as PCC (A23, red arrow), PPC (A39, white arrow) and mPFC (A9 and A10, yellow arrow). Even under this condition, we were able to observe the FPN and DMN ICA components (Figures 5B, C). However, compared with the human resting-state DMN component (Figure 1B), the activity in PCC (A23) and the inferior part of PPC (A39) became less apparent, probably due to task-induced deactivation. Based on our fingerprint analysis, the resting marmoset component Comp-B was the closest to this human DMN component (Figure 5C) over other resting/task human components. Thus, the sub-cortical activity of the resting marmoset Comp-B may be closer to that of the deactivated human DMN, or that a human-like activated DMN may not really be the default mode for the marmoset.

## 4 Discussion

The DMN has a characteristic shape (Supplementary Figure 2), such as well separated PCC, PPC and dlPFC regions, but defining it in the marmoset remains a challenge. Based on anatomy, Brodmann's areas vary widely across primate species. In particular, the ratio of the size of the visual cortex to the total cortex varies greatly (Rosa and Tweedale, 2005; Fukushima et al., 2019), and the remaining sensory, motor, and functional cortex are generally more to the anterior side in the common marmoset. For this reason, the A23 and A31 cortical areas (except A23V) are much more anterior than in the human. Although the PCC and PPC regions are located posterior in the human, these regions of the marmoset are not always in a posterior location (see Figure 1, which shows the human and marmoset A23 and A31 locations). As a result of neuroanatomical verification (Figure 1), auditory based task-induced deactivation (Figure 2), and fingerprint analysis by sub-cortical regions (Figure 4), we determined that Comp-A is the FPN and Comp-B is the DMN in the common marmoset brain. Comp-A has been mentioned in various papers with a debate over it being the DMN or the FPN (Belcher et al., 2013; Ghahremani et al., 2017; Liu et al., 2019; Schaeffer et al., 2019; Hori et al., 2020; Garin et al., 2022; Ngo et al., 2023) and we see several pieces of evidence that suggest it might be the DMN, such as part of MIP showing task-induced deactivation (Figure 2D), A23V showing task-induced deactivation in macaque monkeys (Mantini et al., 2011), and fingerprint analysis between the resting human DMN and resting marmoset Comp-A being closer than with Comp-B. Although we propose that Comp-B is more suitable for the DMN component through several lines of evidence, further investigation will be required to definitively determine the DMN in the common marmoset brain.

Our results suggest that the structure of large-scale brain network components of the human, such as the DMN, should not always be relied upon for defining the equivalents in species such as the common marmoset. The human brain is much larger than that of the marmoset (190-fold difference in weight Van Essen et al., 2019) and is gyrified with deep sulci. Due to this the BOLD (blood oxygenation level-dependent) signal is well separated between regions. For example, Supplementary Figure 4 shows a human SMN component with a clear boundary around the somatomotor (A4) and somatosensory cortex (A1/2, 3). However, the marmoset cortex is smooth and relatively small, and Comp-B showed ambiguity in regional boundaries around PE, A1/2, A3, and A4ab, even in ultrahigh field (9.4T) fMRI data. The temporal resolution of fMRI scans of the marmoset brain is low compared with the human. Current marmoset data has TR = 2.0 s and group ICA was obtained from 140 frames × 48 sessions. This small dataset may result in insufficient component decomposition. A higher temporal resolution (or larger frame number) and larger session data would be required to correct this ambiguity for the marmoset.

Garin et al. (2022) also showed the differences between the human DMN and the non-human primate FPN (Comp-A) in resting state fMRI using fingerprint analysis. They showed that the human PCC highly correlated with the PPC and the mPFC, but the non-human primate PCC is highly correlated with the PPC and the dlPFC. Furthermore, the human mPFC highly correlated with PPC, Temp, and PCC, but the non-human primate PCC does not. However, marmoset FPN component was originally named by Ghahremani et al. (2017) based on seed analysis of superior colliculus, and their claim was supplanted by more direct evidence from visual-fixation task-induced deactivation of the DMN (Liu et al., 2019). Thus, Garin et al. (2022) could not exclude either the front temporal network (FTN) or the FPN as homologous candidates to the human DMN. We directory challenged this issue, and based on auditory task-induced deactivation we found Comp-B is more suitable as the DMN, and based on fingerprint analysis, Comp-A is closer to the (task-induced activated) human FPN. In our study, we could not find a clear FTN component by group ICA, and multiseed-based connectivity analysis of the marmoset mPFC region did not show strong connectivity with the temporal lobe (in comparison to the human case) (Figure 3). Therefore, no judgment can be made regarding this network and further research is required.

The function of the DMN in the resting marmoset also resulted in questionable results as to whether it is homologous to the resting human DMN. Although marmosets might not be resting in the same manner as a human during fMRI experiments, marmosets are usually trained to become familiar with the MRI machine, and in our experiment four marmosets went through as many as 12 sessions. This training to be familiar allows us to say that the marmoset can be considered to be in a state of rest. However, we found that the resting marmoset DMN component (Comp-B) was not close to the resting human DMN component; based on fingerprint analysis we found that it was closer to the wm-task human DMN component. The human DMN was highly suppressed in this task (Figure 5A) and fingerprint analysis showed a low correlation in activity between the DMN component and sub-cortical voxels (Figure 4D). Conversely, activity in the resting human DMN correlated highly with sub-cortical voxels, and marmosets appear to have correlations somewhere in-between. Thus, the DMN of the marmoset (Comp-B) is not as active as the human in the resting state, and it implies that we should consider that a marmoset does not reach a state that could be considered the default mode for the human. The human DMN appears to have an advanced function (Binder, 2012) that may be underdeveloped in non-human primates (such as the common marmoset) (Garin et al., 2022). From now on, we need to consider that the human default mode network is not the same as the default mode for the common marmoset, and possibly for other non-human primates (Garin et al., 2022) and rodent species.

## Data availability statement

Publicly available datasets were analyzed in this study. This data can be found at: Pre-processed auditory task fMRI data of the common marmoset is available at https://doi.org/10.5281/zenodo.7827225.

## Ethics statement

Ethical approval was not required for the study involving humans in accordance with the local legislation and institutional requirements. Written informed consent to participate in this study was not required from the participants or the participants' legal guardians/next of kin in accordance with the national legislation and the institutional requirements. The animal study was approved by the Experimental Animal Committee of RIKEN. The study was conducted in accordance with the local legislation and institutional requirements.

## Author contributions

TO: Conceptualization, Data curation, Formal analysis, Investigation, Software, Writing—original draft, Writing—review & editing. NI: Supervision, Writing—review & editing. AW: Funding acquisition, Supervision, Writing—review & editing.
