# Supplementary material for: A reappraisal of the default mode and frontoparietal networks in the common marmoset brain
Source: Front Neuroimaging. 2024 Jan 9;2:1345643. doi: 10.3389/fnimg.2023.1345643 (PMC10803424; doi:10.3389/fnimg.2023.1345643)
Supplement: Supplementary file 1 [file Data_Sheet_1.DOCX]

Supplementary Material

# Supplementary Figures

Supplementary Fig. 1 | Sub-cortical ROIs of human and marmoset for fingerprint analysis. a, Sub-cortical ROIs of human brain from right. b, Sub-cortical ROIs of human brain from inferior (only right side is presented). c, Sub-cortical ROIs of marmoset brain from right. d, Sub-cortical ROIs of marmoset brain from inferior (only right side is presented).

Supplementary Fig. 2 | Shape of DMN component. (left) marmoset component-A and its shape in the horizontal plane. (center) human DMN component and its shape in the horizontal plane. (right) marmoset component-B and its shape in the horizontal plane.

Supplementary Fig. 3 | Fingerprint analysis result between awake resting marmoset and resting/tasking human ICA components. a, Right cortex of a marmoset surface image. (top) lateral side, (bottom) medial side. Extra components of awake marmoset ICA are mapped onto the brain surface. Z-score range is 3 to 30 for positive, -3 to -30 for negative. b, Fingerprint distance results between awake resting marmoset components (extra version) and resting/tasking human components. White asterisks show the closest components from Comp-A and Comp-B. Marmoset FPN, SAN and pVIS show a similar tendency. Marmoset DMN and PMN show a similar tendency.

Supplementary Fig. 4 | Human brain other network components. a, Right cortical surface of the human brain. Human resting-state network components are mapped onto the brain surface. Z-score range is 2 to 8 for positive, -2 to -8 for negative.

Supplementary Table 1. GLM analysis results of awake marmoset passive auditory task-fMRI

| ROI name (left cortex) | significant voxel rate | significant voxel num | ROI voxel num | mean of significant voxels |  | ROI name (right cortex) | significant voxel rate | significant voxel num | ROI voxel num | mean of significant voxels |
| --- | --- | --- | --- | --- | --- | --- | --- | --- | --- | --- |
| PE | 4.21% | 11 | 261 | -4.89 |  | PE | 24.72% | 66 | 267 | -4.90 |
| PEC | 16.18% | 11 | 68 | -5.41 |  | PEC | 57.35% | 39 | 68 | -4.88 |
| PF | 0% | 0 | 47 | NaN |  | PF | 0% | 0 | 45 | NaN |
| PFG | 53.33% | 32 | 60 | -4.94 |  | PFG | 0% | 0 | 64 | NaN |
| PG | 32.05% | 25 | 78 | -4.94 |  | PG | 0% | 0 | 76 | NaN |
| LIP | 2.31% | 3 | 130 | -4.55 |  | LIP | 2.42% | 3 | 124 | -4.91 |
| MIP | 0% | 0 | 69 | NaN |  | MIP | 23.68% | 18 | 76 | -5.14 |
| VIP | 0% | 0 | 27 | NaN |  | VIP | 23.08% | 6 | 26 | -5.58 |
| A23a | 0% | 0 | 73 | NaN |  | A23a | 0% | 0 | 66 | NaN |
| A23b | 7.35% | 10 | 136 | -4.72 |  | A23b | 0% | 0 | 137 | NaN |
| A31 | 23.08% | 12 | 52 | -5.32 |  | A31 | 12.28% | 7 | 57 | -5.05 |
| PGM | 0% | 0 | 83 | NaN |  | PGM | 2.50% | 2 | 80 | -4.43 |
| A19M | 0% | 0 | 99 | NaN |  | A19M | 15.69% | 16 | 102 | -5.21 |
